# Leaf Vein-Inspired Hierarchical Point–Line–Plane BNNT/WS_2_/Ag Welded Network for Efficient Heat Dissipation

**DOI:** 10.3390/nano16211368

**Published:** 2026-10-27

**Authors:** Changzeng Yan, Xiang Li, Zeyu Dong, Guiying Li, Xiuhua Ma, Yuhan Zhang, Guangqi Bai, Dae Joon Kang

**Affiliations:** 1 Nano-Micro Energy Catalysis Lab, College of Smart Energy, Shanghai Jiao Tong University, Shanghai 200240, China; lx0206@sjtu.edu.cn (X.L.); wngwnga0@sjtu.edu.cn (Z.D.); liguiying@sjtu.edu.cn (G.L.); maxiuhua0409@sjtu.edu.cn (X.M.); wdmzszyh@sjtu.edu.cn (Y.Z.); bgq3106294962@sjtu.edu.cn (G.B.); 2 Department of Physics, Sungkyunkwan University, 2066, Seobu-ro, Jangan-gu, Suwon 16419, Republic of Korea; djkang@skku.edu

**Keywords:** BNNT, WS_2_ nanosheet, silver nanoparticle, thermal filler, interfacial thermal resistance, nanocomposite

## Abstract

Severe heat buildup within chips limits device stability and service life, and thermally conductive yet electrically insulating polymer nanocomposites become critical thermal interface materials (TIMs) for electronics cooling. The intrinsic interfacial thermal resistance (ITR) between discrete thermally conductive fillers in TIMs remains the core bottleneck limiting thermal conductivity improvement, and rationally constructing three-dimensional (3D) interconnected filler networks represents a feasible and efficient strategy to address this issue. Inspired by the natural hierarchical point–line–plane topological skeleton of a plant leaf, which realizes efficient internal matter transfer via long spicular lines, discoid planar scaffolds, and vein nodes, we design and produce a novel BNNT/WS_2_/Ag ternary hybrid thermally conductive network within an epoxy matrix. Specifically, high aspect ratio boron nitride nanotubes (BNNTs) act as one-dimensional linear thermal conduction skeletons mimicking leaf veins, while tungsten disulfide (WS_2_) nanosheets serve as two-dimensional planar heat-collecting and heat-spreading scaffolds with large specific surface areas. Notably, in situ photodeposited silver (Ag) nanoparticles function as biomimetic vein nodes between adjacent fillers. Low-temperature sintering enables these Ag nodes to tightly connect BNNT linear skeletons and WS_2_ planar nanosheets, effectively bridging isolated thermal conductive units, which is expected to reduce the effective thermal resistance of the composite system. This hierarchical point–line–plane structure constructs a continuous, unobstructed 3D thermal conduction highway inside the epoxy matrix. The resultant BNNT/WS_2_/Ag/epoxy nanocomposite exhibits excellent thermal conduction performance, with a prominent in-plane thermal conductivity of 21.5 W m^−1^ K^−1^ and a high through-plane thermal conductivity of 7.63 W m^−1^ K^−1^. Benefiting from the hierarchical topological structure and interconnected conductive network, the as-prepared nanocomposites possess outstanding heat dissipation capability and hold great promise for high-efficiency thermal management in advanced electronic devices.

## 1. Introduction

The continuous miniaturization, integration, and increasing power density of modern electronic devices have imposed increasingly stringent requirements on thermal management materials [1,2]. As heat generation becomes increasingly concentrated at the device level, efficient dissipation of localized heat is critical for maintaining operational stability and reliability [3,4]. Polymer-based TIMs are particularly attractive for electronic packaging because of their low density, good processability, flexibility, and electrical insulation. Nature provides valuable design inspiration for constructing efficient mass and heat transport networks. Typical inspiration comes from hierarchically interconnected venation systems of plant leaves. Primary veins construct long-range transport pathways. Branched veins distribute fluxes across large planar regions. Plenty of interconnected nodes preserve the overall network continuity. Such a hierarchical architecture offers an appealing structural concept for designing high-performance TIMs, which likewise require uninterrupted long-distance heat conduction pathways, broad lateral heat-spreading planes, and robust interconnections between discrete conductive building blocks. Nevertheless, pristine polymers exhibit intrinsically low thermal conductivity (typically 0.1–0.5 W m^−1^ K^−1^), which severely limits their practical heat dissipation capacity [5].

To address this drawback, highly thermally conductive fillers including metal particles [6], ceramics [7], graphene [8,9,10], carbon nanotubes [8,11], boron nitride [11,12,13,14,15] and MXenes [16] have been incorporated into polymer matrices to boost macroscopic thermal transport. Unfortunately, high intrinsic thermal conductivity of individual fillers cannot be directly translated into superior composite heat dissipation performance. Randomly dispersed fillers are frequently isolated by low thermal conductivity polymer regions, and poor filler–filler contacts create numerous thermally resistive interfaces. Heat flow must repeatedly cross these interfaces during transport, resulting in substantial ITR, interrupted heat-transfer pathways, and localized thermal bottlenecks. Although increasing filler loading raises the probability of filler contact, excessive loading inevitably triggers filler aggregation, introduces extra heterogeneous interfaces, and deteriorates composite processability [17]. Accordingly, a major scientific challenge lies in transferring the hierarchical connectivity principle of natural transport networks into polymer TIMs, and assembling isolated conductive fillers into a continuous three-dimensional (3D) heat-transfer network with minimized ITR [18].

**Figure 1:**
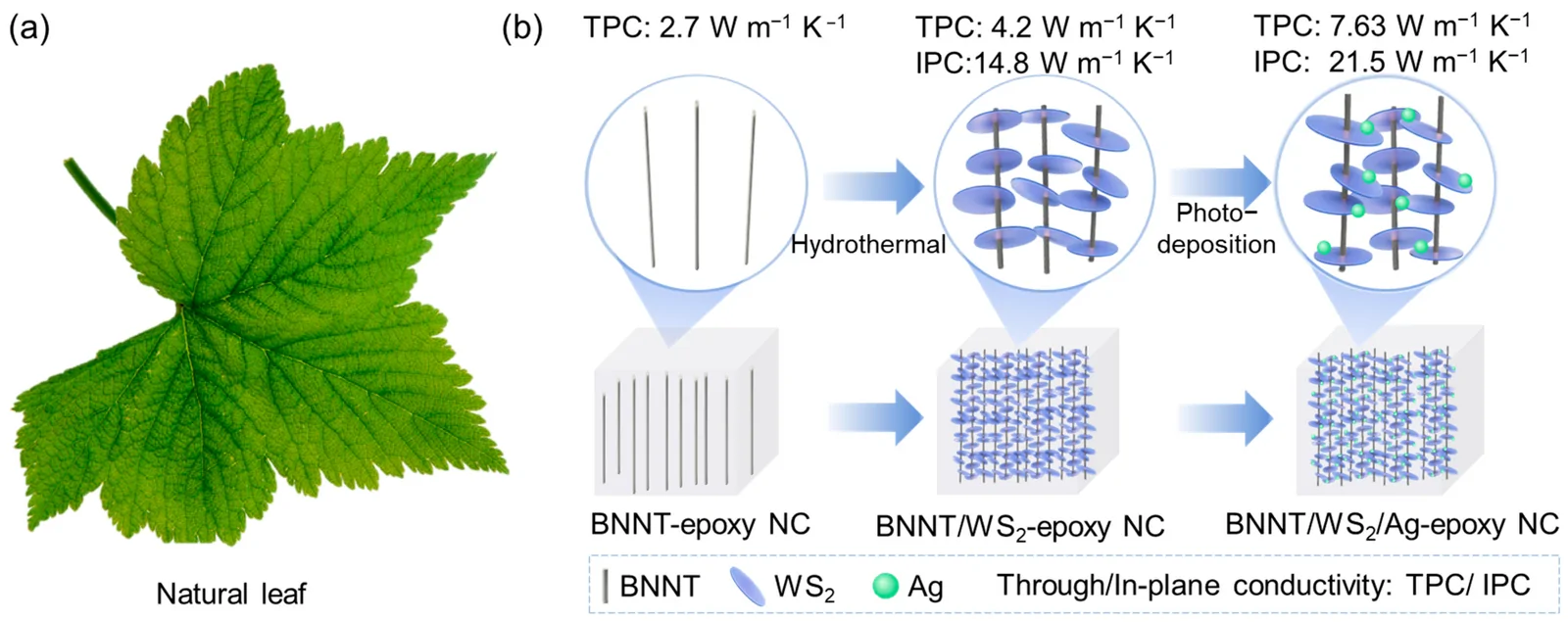
(**a**) A photo of a leaf; (**b**) scheme of preparing the BNNT, BNNT/WS_2_, BNNT/WS_2_/Ag nanoheterostructure fillers and their corresponding epoxy nanocomposites.

Natural vein structures provide a feasible solution to this dilemma (Figure 1a). Plant leaf venation forms hierarchical interconnected networks. Primary veins realize long-range transport. Progressively branched veins spread fluxes over the 2D leaf surface. Numerous junctions maintain the continuity of the whole network [19]. Rather than relying on isolated single pathways, this hierarchical design integrates long-range transport, large-area heat distribution and multidirectional interconnection within an integrated network. From the perspective of thermal network engineering, this structural principle is particularly attractive: continuous skeletons provide rapid long-distance heat conduction, extended planar structures redistribute heat over larger areas, and interparticle junctions bridge neighboring transport pathways. Such a vein-inspired hierarchical network is promising for mitigating structural discontinuity between conductive fillers and suppressing ITR originating from isolated conductive domains. Related hierarchical cross-interface transport concepts have also been reported in the recent literature. Here, the leaf vein analogy is used as a qualitative design inspiration, rather than an exact quantitative reproduction of natural venation structures.

Realizing this biomimetic strategy in polymer composites requires conductive building blocks with complementary dimensional characteristics. Existing approaches, such as self-assembly, filler alignment, electrospinning, and ice templating, have proven that controlling filler organization can substantially improve network connectivity [20]. However, single-component low-dimensional fillers rarely satisfy all structural requirements for efficient 3D thermal conduction. One-dimensional (1D) fillers provide elongated conductive pathways but have limited lateral contact areas. 2D nanosheets provide broad heat-spreading surfaces, although contact resistance can remain high between adjacent sheets. Strategically deployed zero-dimensional (0D) conductive particles can serve as connecting nodes at these interfaces to link discrete conductive units. This dimensional complementarity enables the integration of point, line and plane motifs into hierarchical architectures, in which individual low-dimensional blocks are progressively interconnected into a continuous 3D thermal network [21,22].

Against this background, BNNTs are selected as “line” units for long-range heat conduction. Analogous to carbon nanotubes, BNNTs are promising electrically insulating thermal fillers featuring a high aspect ratio, a large specific surface area, superior mechanical strength, excellent chemical and oxidation stability, a wide band gap (ca. 5.9 eV), and the high intrinsic thermal conductivity (300–2000 W m^−1^ K^−1^) [23,24]. These remarkable characteristics have stimulated extensive interest in BNNT-reinforced polymer composites for thermal management applications.

Transition-metal dichalcogenides (TMDs) also display attractive thermal transport characteristics and can provide large 2D contact areas [25]. Among them, WS_2_ exhibits the best room temperature thermal conductivity of 142 W m^−1^ K^−1^, outperforming MoS_2_ (103 W m^−1^ K^−1^), MoSe_2_ (54 W m^−1^ K^−1^), and WSe_2_ (53 W m^−1^ K^−1^) [26]. WS_2_ nanosheets act as “plane” components, extending linear conductive pathways into broader heat-transfer interfaces. Introducing metal nanoparticles into hybrid filler systems further strengthens interparticle coupling and improves thermal transport. Additionally, due to the nanoparticle size effect, metal nanoparticles realize relatively low-temperature welding (appr. 200 °C) and possess self-repairing properties [18]. Accordingly, Ag nanoparticles function not merely as thermally conductive additives, but more importantly as welding points that connect neighboring line–plane building blocks, reduce contact thermal resistance, and complete the 3D thermal network.

Inspired by the above biomimetic design philosophy, we fabricate a hierarchical point–line–plane BNNT/WS_2_/Ag thermal network in an epoxy matrix in the present work. BNNTs constitute the primary 1D skeleton; WS_2_ nanosheets enlarge the heat-collection area and establish lateral bridges between neighboring skeletons; and Ag nanoparticles function as low-temperature welding nodes formed during the staged epoxy-curing process. The Ag nanoparticles act as interconnectors between neighboring BNNT/WS_2_ units, promoting the formation of an interconnected 3D thermally conductive network (Figure 1b) and reducing filler–filler contact resistance. At a total filler loading of 15.4 wt%, infrared thermal imaging of the resulting BNNT/WS_2_/Ag-epoxy composite confirms its enhanced heat-transfer capability, highlighting the great potential of this hierarchical network design for developing high-performance polymer-based thermal management materials. The key structural advantage lies in integrating long-range BNNT pathways, 2D WS_2_ heat-spreading planes, and Ag-assisted junction bridging within one network. Specifically, BNNTs provide long-range 1D heat conduction skeletons, WS_2_ nanosheets enlarge lateral heat-spreading interfaces, and Ag nanoparticles bridge neighboring units to improve network continuity.

## 2. Materials and Methods

### 2.1. Source of Materials

Methylhexahydrophthalic anhydride (CAS No. 25550-51-0, 98%), tungsten chloride (CAS No. 13283-01-7, WCl_6_, metals basis, Sigma-Aldrich (Saint Louis, MO, USA), ≥99.9%), thioacetamide (CH_3_CSNH_2_, Sigma-Aldrich, ≥99.0%), and epoxy resin E-51 (CAS No. RH-01221) were purchased from Tansoole one-stop shopping platform (Shanghai, China). Silver nitrate (CAS No. 7783-99-5, 99%), sulfuric acid (H_2_SO_4_, CAS No. 7664-93-9, 70%), hydrogen peroxide solution (H_2_O_2_, 7722-84-1, AR, 30 wt% in H_2_O) and ammonia (25% solution) were purchased from Aladdin Co., Ltd. (Shanghai, China). BNNT powder (CAS No. 7440-42-8) with average diameter ~50 nm was purchased from Shanghai AIXPENG Materials Technology Co., Ltd. (Shanghai, China). The above reagents were utilized without any further purification except for the BNNT powder.

### 2.2. Materials

#### 2.2.1. Synthesis of BNNT/WS_2_, and BNNT/WS_2_/Ag Nanoheterostructure Fillers

Surface modification of BNNTs: Prior to the utilization, the purchased BNNTs were dispersed in 5 mL H_2_O_2_, followed by the dropwise addition of 10 mL H_2_SO_4_ and further stirring for 2 h to make them better hydrophilic, then the product was washed to neutral with deionized water, collected using a filtration technique, and finally dried adequately in a vacuum oven at 80 °C for 12 h.

Hydrothermal synthesis of BNNT/WS_2_: Typically, the above surface-modified BNNTs were dispersed ultrasonically in a 50 mL Teflon-lined autoclave containing a 40 mL aqueous solution of 1.19 g of WCl_6_ and 2.28 g of CH_3_CSNH_2_. After magnetic stirring for 1 h, the autoclave was heated and maintained at 260 °C for 24 h, and then allowed to cool to room temperature naturally. The received BNNT/WS_2_ nanoheterostructures were washed with deionized water and ethanol thoroughly to remove the residuals, and finally dried in a vacuum oven at 50 °C for 6 h.

Photo-synthesis of BNNT/WS_2_/Ag: 0.8 g of BNNT/WS_2_ nanoheterostructures was dispersed in 50 mL of 0.5 M silver nitrate aqueous solution and stirred for 0.5 h. The mixture solution was irradiated with a Xenon lamp (CEL-HXF300-T3, CEAULIGHT, Beijing, China) for 10 min to decorate the Ag nanoparticles on the WS_2_ nanosheets. During the photochemical reduction procedure, the solution temperature was kept at 5 °C. Eventually, the collected dark-gray product was washed thoroughly with ethanol, then dried completely at 80 °C for 12 h in a vacuum oven.

#### 2.2.2. Fabrication of BNNT, BNNT/WS_2_, and BNNT/WS_2_/Ag-Epoxy Nanocomposites

The prepared BNNT, BNNT/WS_2_, and BNNT/WS_2_/Ag fine powder nanofillers were placed in three individual Teflon molds, thoroughly mixed with a mixture solution of epoxy resin (E-51), curing agent (methylhexahydrophthalic anhydride) and catalyst (N,N-dimethylbenzylamine). After that, these mixtures with different filler loading ratios (5.1 wt%, 10.7 wt%, 15.4 wt%, and 20.6 wt%) were placed in an evacuated oven for 6 h for degassing. Finally, all samples were cured successively, 120 °C for 2 h, 160 °C for 2 h, and 200 °C for 2 h to obtain the thermal interface material films for further study.

### 2.3. Methods

The morphologies of the fillers and composite fracture/cross-sectional surfaces were examined by field-emission scanning electron microscopy (FE-SEM, Regulus 8100, Hitachi High-Technologies Corporation, Tokyo, Japan) with an accelerating voltage of 15 kV. The microscopic morphology of BNNT, BNNT/WS_2_, and BNNT/WS_2_/Ag was observed using a Transmission Electron Microscope (TEM, FEI spirit T12, Hitachi High-Technologies Corporation, Tokyo, Japan). X-ray diffraction (XRD, D8 Advance, Bruker AXS GmbH, Karlsruhe, Germany) was used to measure the crystal structure of the prepared sample, and the 2θ value measured by XRD was in the range of 10–80°, and the test was performed with a sweep speed of 0.2 s and a step size of 0.02. The thermal diffusivity (D, mm^2^/s) was measured by a laser flash method (LFA 467, NETZSCH-Gerätebau GmbH, Selb, Germany). The density (ρ, g/cm) was evaluated by a water displacement method. The specific heat of the nanocomposite was measured by differential scanning calorimetry DSC (NETZSCH 200 F3, NETZSCH-Gerätebau GmbH, Selb, Germany) using a heating rate of 20 °C/min under nitrogen at a temperature of −20 to 250 °C (Cp, J)/(g·K)). The thermal conductivity λ (W m^−1^ K^−1^) is calculated by the formula λ = ρ × Cp × D. The tensile properties of the composite were measured by an electronic universal testing machine (AG/X Plus 100N, China). A resistivity tester (EC-80P, Nittoseiko Co., Ltd., Tokyo, Japan) was used to characterize the electrical conductivity of nanocomposites. Their surface temperature distributions during heat transfer were monitored by the thermal infrared camera (FLIT T450sc, Teledyne FLIR LLC., Wilsonville, OR, USA).

## 3. Discussion

Cross-sectional SEM observations of the BNNT/WS_2_/Ag-epoxy composite (Figure S2) show close contact between the hybrid filler and the epoxy matrix, with no obvious interfacial voids or cracks in the indicated regions. Such intimate interfacial contact is favorable for decreasing interfacial thermal resistance and maintaining continuous heat-transfer pathways across the filler–matrix interface. It should be noted that SEM and TEM characterization merely provide qualitative visual information. Statistical quantification regarding filler orientation, dispersion state, and 3D network connectivity was not performed in the present work.

**Figure 2:**
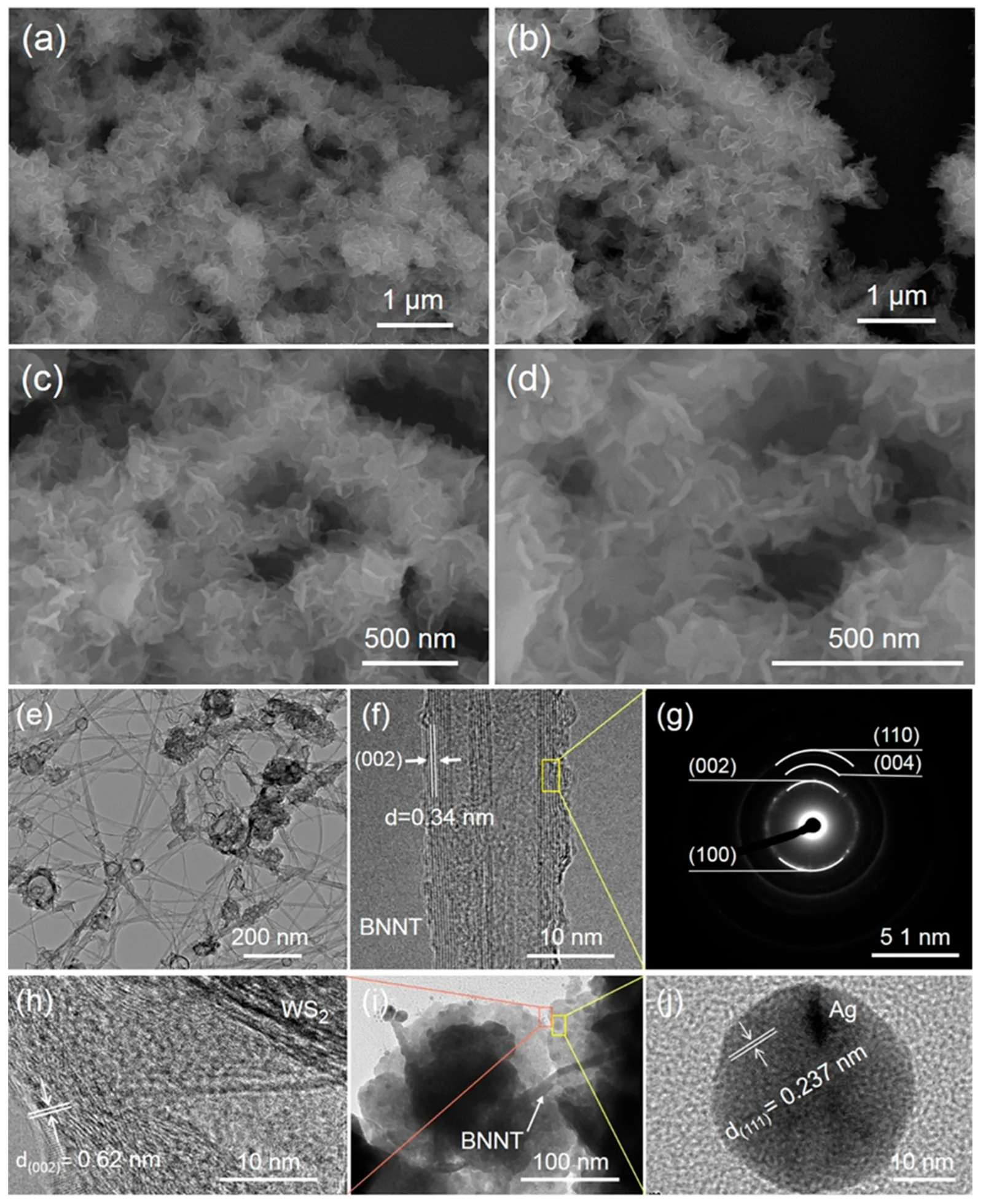
(**a**–**d**) SEM images of the BNNT/WS_2_/Ag nanoheterostructure at different magnifications. (**e**–**g**) TEM, HR-TEM image and SAED pattern of BNNT. (**i**) HR-TEM image of an individual BNNT/WS_2_/Ag nanoheterostructure (Ag content, 19.6 wt%), along with enlarged views of its compositional (**h**) WS_2_ nanosheet and (**j**) Ag nanoparticle.

Figure 2 presents the microscopic morphology of surface-modified BNNT and BNNT/WS_2_/Ag nanoheterostructure fillers. The tight, gap-free interfaces at BNNT-WS_2_-Ag heterojunctions are directly visualized in SEM images of BNNT/WS_2_/Ag nanoheterostructures (Figure 2a–d), and, at high resolution, the BNNTs served as the scaffold or skeleton of the BNNT/WS_2_/Ag nanoheterostructure to string together all the WS_2_ nanoplates decorated with Ag nanoparticles. The hybrid structures have an average apparent diameter of approximately 200 nm and a length of approximately 6 μm. TEM observations of the treated BNNTs (Figure 2e–g) reveal a relatively smooth tubular morphology with a diameter of ca. 15 nm; the corresponding diffraction features indicate that the treated BNNTs contain polycrystalline domains. Figure 2i displays an individual BNNT/WS_2_/Ag nanoheterostructure with clear compositional BNNT, WS_2_ nanosheets, and optimized Ag nanoparticle ratio (19.6 wt%, the highest fraction examined in this series), looking like a kebab seasoned with cumin. The inter-planar d-spacing was estimated to be 0.62 nm by reduced fast Fourier transform (RFFT) in Figure 2h, identical to the (0 0 2) lattice plane of hexagonal WS_2_. In Figure 2j, the deposited Ag nanoparticle has a diameter of approximately 30 nm and exhibits clear lattice fringes with a spacing of 0.237 nm, corresponding to the Ag (1 1 1) plane. The intimate contact between Ag and the BNNT/WS_2_ framework provides physical junctions that can connect neighboring hybrid units during low-temperature sintering, thereby promoting a more continuous 3D heat conduction network and decreasing thermal resistance at filler–filler contacts.

The XRD pattern of the BNNT/WS_2_/Ag nanoheterostructure (Figure S1, Supplementary Information) has two broad peaks around 26.829° and 42.34°, corresponding to their characteristic (0 0 2) and (1 0 1) planes of single-phase hexagonal boron nitride (JCPDS No. 34-0421). Broad diffraction peaks at 2θ values of 12.74°, 33.4°, 46.8°, 57.9°, 66.18°, and 72.34° are in line with the standard pattern of hexagonal WS_2_ (JCPDS No. 87-2417). The (0 0 2) lattice-oriented WS_2_ diffraction lines appear as a predominant peak of the nanosheet form; no impurity diffraction peaks were detected, elucidating a complete transformation from WCl_6_ precursor to highly crystalline WS_2_. The weak peaks located at 2θ of 64.4° and 77.4° are assigned to the (2 2 0) and (3 1 1) planes of face-centered-cubic Ag, respectively (JCPDS No. 04-0783).

**Figure 3:**
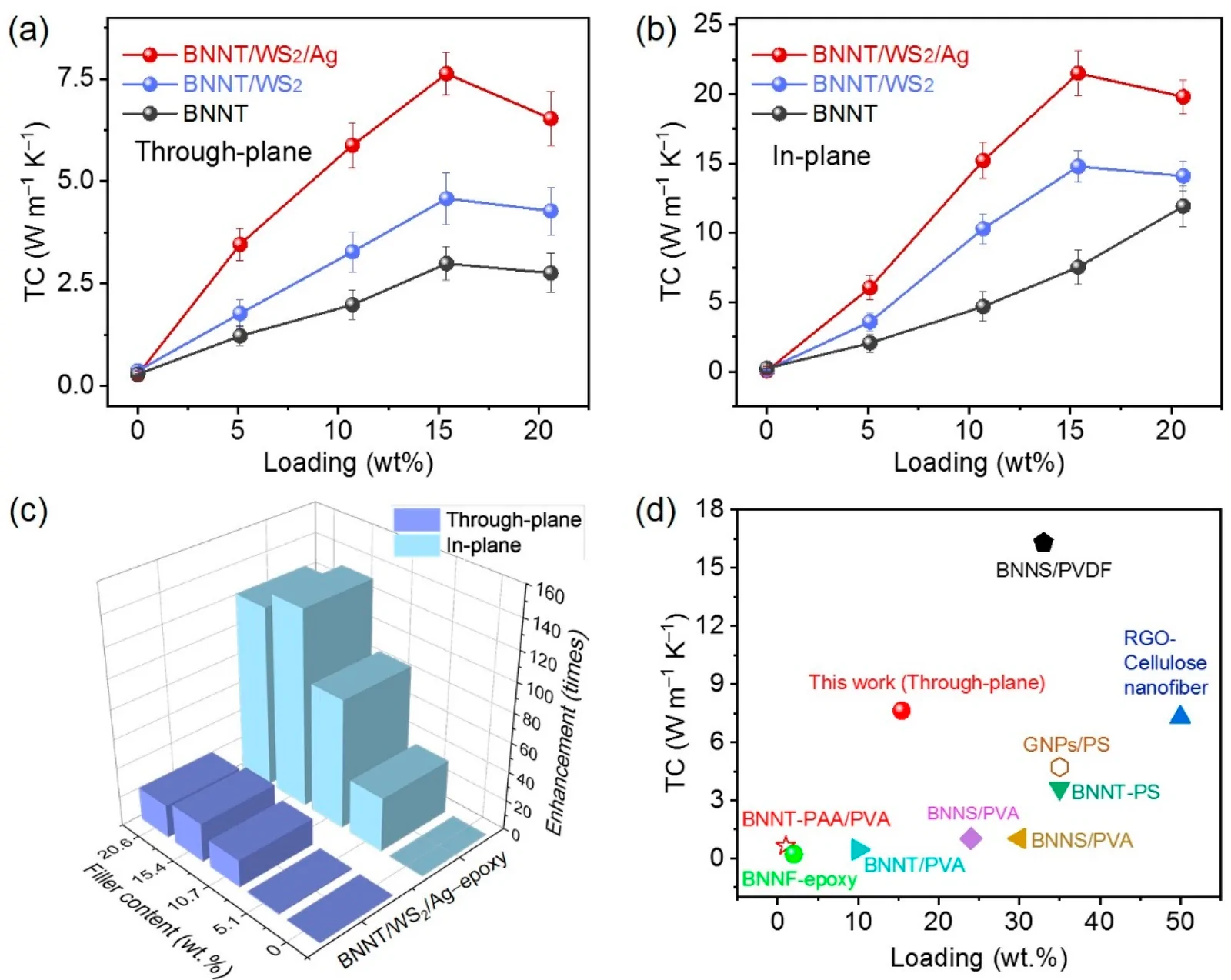
(**a**) Through-plane and (**b**) in-plane thermal conductivity of the BNNT, BNNT/WS_2_, and BNNT/WS_2_/Ag-epoxy nanocomposite films measured at 25 °C; (**c**) enhancement rate of in-plane and through-plane thermal conductivity for BNNT/WS_2_/Ag-epoxy nanocomposites as the filler content increases; (**d**) comparison of the heat dissipation performance of this work with other relevant composites reported elsewhere [12,26,27,28,29,30,31].

Figure 3a,b shows the in-plane and through-plane thermal conductivities of BNNT, BNNT/WS_2_, BNNT/WS_2_/Ag-epoxy nanocomposites. Incorporation of WS_2_ and, particularly, Ag markedly enhances thermal transport in both directions. Specifically, BNNT/WS_2_/Ag-epoxy nanocomposites display not only the best in-plane but also the best through-plane thermal conductivity, because the Ag particles welded the interconnected BNNT/WS_2_ network through a low-temperature sintering process throughout the entire epoxy matrix, which is consistent with reduced effective contact resistance. When the BNNT/WS_2_/Ag filling content reaches 15.4 wt%, the corresponding epoxy nanocomposite has ultra-high in-plane and through-plane thermal conductivities of 21.5 W m^−1^ K^−1^ and 7.63 W m^−1^ K^−1^, which are 26.37 and 133.4 times higher than that of a pure epoxy matrix (Figure 3c). Accordingly, for BNNT and BNNT/WS_2_-epoxy nanocomposites, the thermal contact resistances are 2.27 × 10^6^ K W^−1^ (ca. 6.42 × 10^−9^ m^2^ K W^−1^) and Rc = 1.14 × 10^6^ K W^−1^ (ca. 3.22 × 10^−8^ m^2^ K W^−1^), respectively. The R_c_ value for BNNT/WS_2_/Ag-epoxy nanocomposite obtained from Equation (S3) is 7.7 × 10^5^ K W^−1^ (ca. 2.18 × 10^−8^ m^2^ K W^−1^), which is lower than that of other epoxy nanocomposites (See Supporting Information). We assume Ag nanoparticles deposited on the surface of heat-collecting WS_2_ nanosheets with extremely large specific surface area effectively “weld” all the networked thermally conductive filler, constructing a much lower ITR after sintering at a relatively low melting point; the enhancement in thermal conductivity will be larger. Furthermore, as the filler ratio increases, more heat conduction paths will be produced, which will lead to a greater enhancement in both in-plane and through-plane thermal conductivities. The related BN nanomaterials and polymer-based composites are summarized in Figure 3d [12,26,27,28,29,30,31]. The prepared highly thermally conductive epoxy composite containing rationally designed BNNT/WS_2_/Ag nanoheterostructure filler is superior to the composites with similar BNNT and BNNT/WS_2_ filling ratios, verifying that the networked and interconnected fillers enhance heat transfer more effectively. These resistance values are model-derived effective contact-resistance estimates rather than direct measurements of microscopic thermal-boundary resistance. The intimate BNNT/WS_2_/Ag junctions observed by SEM/TEM therefore provide structural support for the lower model-derived effective contact resistance and the enhanced thermal conductivity. Compared with recent hybrid BNNT/nanosheet and BN/silver network strategies [17,18,19], the present design combines 1D transport, 2D heat-spreading, and metal-assisted junction bridging within a single hierarchical filler network.

**Figure 4:**
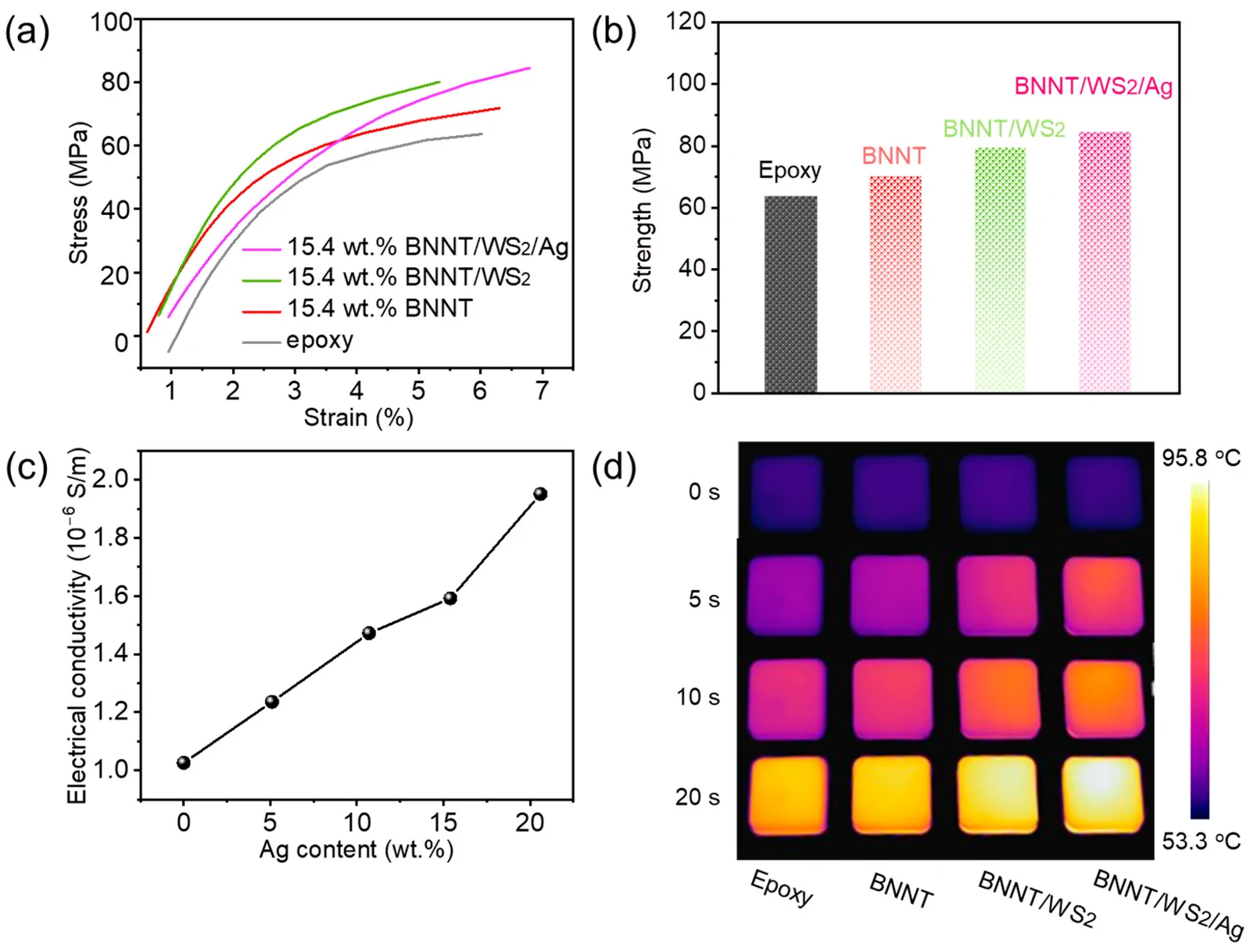
(**a**) Mechanical properties and (**b**) strength curves of BNNT, BNNT/WS_2_, and BNNT/WS_2_/Ag-epoxy nanocomposites at an optimized 15.4 wt% filler loading amount, (**c**) electrical conductivities of BNNT/WS_2_/Ag-epoxy nanocomposite films, (**d**) corresponding infrared thermal images during the heating process.

The mechanical properties of thermal management materials are essential for real portable microelectronics. In this work, the mechanical strength of epoxy resin was enhanced by adding BNNT, BNNT/WS_2_, and BNNT/WS_2_/Ag nanoheterostructure fillers [31]. According to Figure 3, the optimized loading amount of 15.4% BNNT, BNNT/WS_2_, and BNNT/WS_2_/Ag in epoxy resin is selected and investigated. Tensile tests were performed on these nanocomposites using an electronic-type universal testing machine. Figure 4a shows the stress–strain curve of epoxy resins with different fillers. As can be observed from the figure, all specimens undergo immediate failure once the tensile stress reaches its maximum value. The stress–strain curve exhibits distinct non-linear characteristics prior to reaching the maximum stress. The modulus of the epoxy resin continuously increases when filled with BNNT, BNNT/WS_2_, and BNNT/WS_2_/Ag. The tensile strength obtained from the test is plotted in Figure 4b; the tensile strength increase of about 33.07% was observed by adding 15.4 wt% BNNT/WS_2_/Ag. This improvement is attributed to the rigid hybrid filler network and enhanced interfacial mechanical interlocking associated with the nanoscale surface roughness of the Ag-decorated BNNT/WS_2_ structures, which can hinder interfacial sliding and crack propagation under tensile loading.

Figure 4c shows the conductivity of the compressed BNNT/WS_2_/Ag film. As the Ag particle loading increases from 5.1 wt% to 19.6 wt%, one can see that the conductivity increases significantly. Especially when the Ag content is 19.6 wt%, the conductivity is as high as 1.95 × 10^−6^ S/m, which is 1.89 times that of BNNT/WS_2_ (1.03 × 10^−4^ S/m). Although Ag introduces additional electrical pathways, the conductivity remains very low, which is favorable for applications requiring electrical insulation. The thermal-response behavior of the composites was further evaluated by infrared thermography. Neat epoxy and the BNNT, BNNT/WS_2_, and BNNT/WS_2_/Ag-epoxy nanocomposites containing 15.4 wt% filler were placed simultaneously on a hot plate maintained at 95.8 °C, and their surface-temperature evolution was recorded (Figure 4d). To investigate the heat absorption capability, all samples were placed on a heating station (95.8 °C). It can be seen in Figure 4d that the surface temperature of the 7.8 wt% BNNT/WS_2_/Ag-epoxy nanocomposite increases continuously over time at a higher ramping rate. It is worth noting that its surface temperature reaches the same as the heating stage after 20 s, while the other samples are much lower than the heating station, indicating its great potential for thermal management applications. Thus, 19.6 wt% Ag is the selected composition within the tested range. The present thermal-conductivity, mechanical properties, electrical insulation performance, and infrared thermal-response measurements provide a proof-of-concept validation for the proposed network structure. Further systematic measurements including repeated thermal cycling and long-term operation evaluation will be conducted in our follow-up work to comprehensively assess the device level reliability. Taken together, the high thermal conductivity, improved tensile strength, retained low electrical conductivity, and rapid infrared thermal response support the combined heat-transfer efficiency, mechanical integrity, and electrical insulation required for thermal management applications.

## 4. Conclusions

BNNT, BNNT/WS_2_, and BNNT/WS_2_/Ag nanoheterostructure fillers were prepared through hydrothermal growth and photodeposition and subsequently incorporated into an epoxy matrix to construct thermally conductive networks. At a filler loading of 15.4 wt%, the BNNT/WS_2_/Ag-epoxy nanocomposite delivers superior in-plane (21.5 W m^−1^ K^−1^) and through-plane thermal conductivity (7.63 W m^−1^ K^−1^) compared with the other counterparts; its through-plane thermal conductivity is 2.7-fold higher than that of the BNNT-epoxy nanocomposite. The Ag-decorated BNNT/WS_2_ fillers form a crosslinked thermal network throughout the epoxy matrix, which is associated with a model-derived effective contact resistance (ca. 2.18 × 10^−8^ m^2^ K W^−1^) at both filler–filler and filler–resin interfaces. Infrared thermal imaging results show that the BNNT/WS_2_/Ag-epoxy nanocomposite exhibits rapid surface temperature rise, highlighting its great potential as a high-performance thermal management material.

## Data Availability

The original contributions presented in this study are included in the article/Supplementary Materials. Further inquiries can be directed to the corresponding author.

## Supplementary Materials

The following supporting information can be downloaded at https://www.mdpi.com/article/10.3390/nano16211368/s1, Figure S1: XRD pattern of BNNT-WS_2_/Ag nanoheterostructure; Figure S2: Cross-sectional SEM images of BNNT-WS_2_/Ag-epoxy nanocomposite at optimized 15.4 wt% heterostructure filler loading. References [32,33] are cited in the supplementary materials.
