# Supplementary material for: Leaf Vein-Inspired Hierarchical Point–Line–Plane BNNT/WS2/Ag Welded Network for Efficient Heat Dissipation
Source: Nanomaterials (Basel). 2026 Oct 27;16(21):1368. doi: 10.3390/nano16211368 (PMC13765041; doi:10.3390/nano16211368)
Supplement: Supplementary file 1 [file nanomaterials-16-01368-s001.zip › nanomaterials-4609482-supplementary.pdf]

Supporting Information

Leaf-Vein-Inspired Hierarchical Point-Line-Plane BNNT/WS_2/Ag Welded Network for Efficient Heat Dissipation

Changzeng Yan [a,*], Xiang Li a, Guiying Li [a], Zeyu Dong [a], Yuhan Zhang [a], Guangqi Bai [a], Xiuhua Ma [a], and Dae Joon Kang [b]

[a] Nano-Micro Energy Catalysis Lab, College of Smart Energy, Shanghai Jiao Tong University, Shanghai, 200240, China; lx0206@sjtu.edu.cn (X.L.); wngwnga0@sjtu.edu.cn (Z.D.); liguiying@sjtu.edu.cn (G.L.); maxiuhua0409@sjtu.edu.cn (X.M.); wdmzszyh@sjtu.edu.cn (Y.Z.); bgq3106294962@sjtu.edu.cn (G.B.)

[b] Department of Physics, Sungkyunkwan University, 2066, Seobu-ro, Jangan-gu, Suwon, 16419, Republic of Korea; djkang@skku.edu

[*]Corresponding author.

E-mail address: czyan@sjtu.edu.cn

Contents:

Fig. S1 XRD pattern of BNNT-WS_2/Ag nanoheterostructure

Fig. S2 Cross-sectional SEM images of BNNT-WS_2/Ag-epoxy nanocomposite at optimized 15.4 wt% heterostructure filler loading

ITR calculations

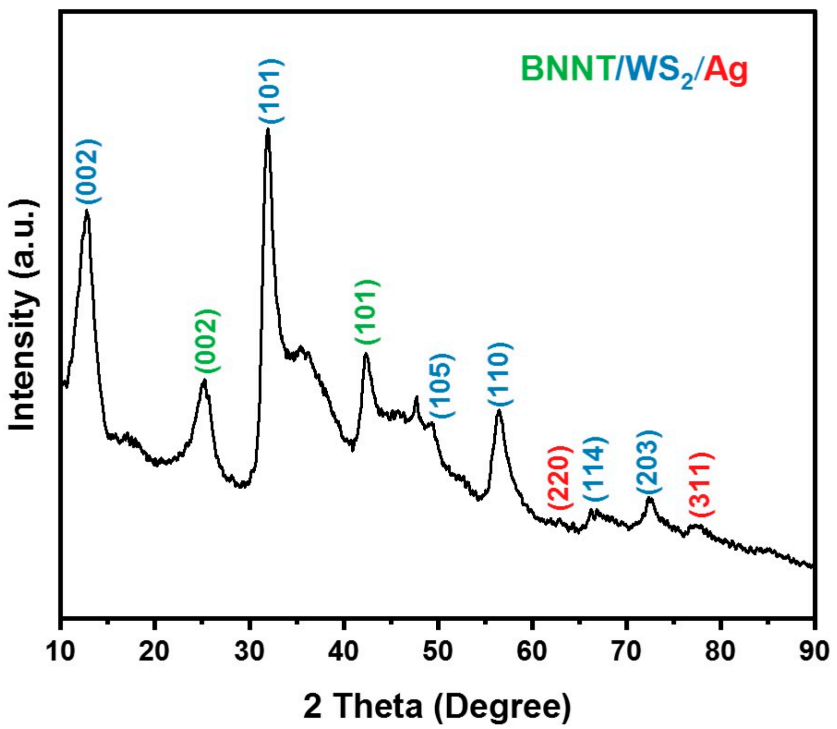

Figure S1 XRD pattern of BNNT-WS_2/Ag nanoheterostructure.

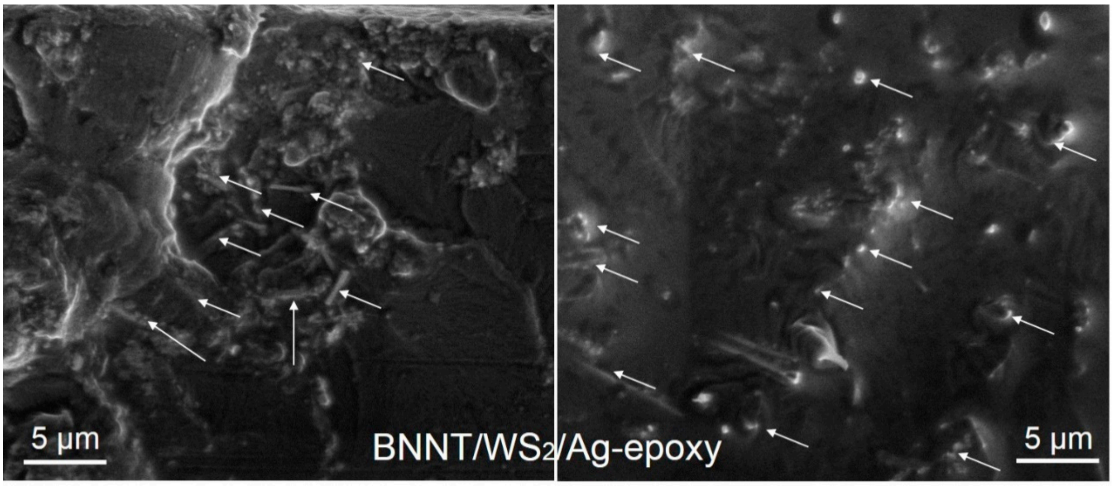

Figure S2 Cross-sectional SEM images of BNNT-WS_2/Ag-epoxy nanocomposite at optimized 15.4 wt% heterostructure filler loading

ITR calculations

The ITR in between the fillers and epoxy matrix are investigated experimentally to further illustrate the exact thermal conductive mechanism. Herein, Monte Carlo model [32] established by Foygelis is applied to our experimental values of *K* for BNNT-epoxy and BNNT/WS_2/Ag-epoxy nanocomposites (equation 1). This model is based on fillers' random distribution and percolating networks in matrix. The geometry of the junction formed among BNNT is emphasized to analyze nanoscale interfacial thermal physics across two BNNT individuals bridged by WS_2. The thermal conductivity of epoxy nanocomposites can be described as the following function of V_f, which is represented by equation [33] (2):

$$V_c(a \gg 1) = \frac{0.6}{a} \quad \text{(S1)}$$

$$\mathrm{K}(V_f, \alpha) = K_0(\mathrm{V}_f - \mathrm{V}_c)^{t(\alpha)} \quad \text{(S2)}$$

Where K_0 is a pre-exponential factor depending on the thermal conductivity of the contacting fillers or the effective thermal conductivity of the filler networks. V_c is the critical volume fraction of the thermal percolation threshold, and $t(\alpha)$ is a conductivity

exponent dependent on the aspect ratio (α=400) of the filler. According to equation (S1), the value of V_c (0.0015) was obtained. Then we use equation (S2) to fit the experimental data with fitted parameters K_0 and $t(\alpha)$, the pre-exponential factor K_0 in equation (2) is estimated to be K_0 = 144 W m^{-1} K^{-1}. The fitting follows most of the experimental data points, especially at high loading ratios. The thermal contact resistance (R_c) between loading materials and epoxy resin matrix is defined as:

$$R_c = (K_0 L V_c^{t(\alpha)})^{-1} \tag{S3}$$

According to this equation, for BNNT and BNNT/WS_2-epoxy nanocomposites, the thermal contact resistances are 2.27 × 10^6 K W^{-1} (*ca.* 6.42 × 10^{-9} m^2 K W^{-1}) and R_c = 1.14 × 10^6 K W^{-1} (*ca.* 3.22 × 10^{-8} m^2 K W^{-1}), respectively. The R_c value for BNNT/WS_2/Ag-epoxy nanocomposite obtained from equation (S3) is 7.7 × 10^5 K W^{-1} (*ca.* 2.18 × 10^{-8} m^2 K W^{-1}) which is lower than other epoxy nanocomposites.
